# COVID-19 vaccine compliance among pregnant women in Poland during the pandemic

**DOI:** 10.3389/fpubh.2026.1917171

**Published:** 2026-09-24

**Authors:** Katarzyna Monika Hinca, Joanna Maria Jassem-Bobowicz, Iwona Jańczewska, Izabela Drążkowska, Iwona Domżalska-Popadiuk, Jolanta Wierzba

**Affiliations:** 1 Division of Neonatology, Medical University of Gdańsk, Gdańsk, Poland; 2 Department of Neonatology, University Clinical Center in Gdańsk, Gdańsk, Poland; 3 Division of Internal and Pediatric Nursing, Medical University of Gdańsk, Gdańsk, Poland

**Keywords:** COVID-19, education, pregnancy, prophylaxis, vaccines

## Abstract

**Introduction:**

This study aimed to assess COVID-19 vaccination adherence and identify the factors associated with vaccine uptake among pregnant women in Poland during the SARS-CoV-2 pandemic. Given the high risk of severe COVID-19 complications, including acute respiratory failure, multiple organ failure, and maternal and neonatal mortality, understanding the factors that influence adherence to immunization guidelines is critical for optimizing maternal–fetal public health strategies.

**Methods:**

This cross-sectional study analyzed 675 questionnaires collected from women hospitalized in the Department of Neonatology at the University Clinical Center in Gdańsk between November 2021 and February 2022. The analysis evaluated associations between vaccination status and vaccine type and demographic characteristics, place of residence, and obstetric history, including fertility disorders and prior miscarriages.

**Results:**

Data revealed that more than 50% of respondents received the COVID-19 vaccine during pregnancy. Multivariable analyses showed that lower educational attainment was the most consistent factor associated with lower odds of vaccination. Older age and residence in a large city were associated with higher odds of receving the vaccine in selected models, whereas obstetric history was not significantly associated with vaccination status.

**Conclusion:**

While vaccine uptake was moderate, the findings highlight a significant demographic divide. These findings suggest that tailored, evidence-based vaccine communication during antenatal care is particularly relevant for women with lower educational attainment.

## Introduction

The World Health Organization (WHO) declared the coronavirus disease 2019 (COVID-19) pandemic on 11 March 2020 (1). The virus was first identified in Wuhan, China, where clinicians first encountered infections caused by a novel coronavirus in November 2019 (2). The distinction from other coronavirus infections was clear—the novel disease was markedly more transmissible and rapidly led to respiratory failure, multiple organ failure, and death in vulnerable individuals (3). Like other viral infections, COVID-19 spread swiftly and quietly around the world, which caused global anxiety (4). Early in 2020, global efforts accelerated to manufacture a vaccine against severe acute respiratory syndrome coronavirus 2 (SARS-CoV-2). In December 2020, both the Pfizer/BioNTech and Moderna vaccines received approval for general use in Europe and the United States (5). These two manufacturers developed an innovative vaccine composed of nucleoside-modified mRNA that encodes a mutated version of the full-length spike protein of SARS-CoV-2, encapsulated in lipid nanoparticles (5, 6). Concurrently, numerous other pharmaceutical companies worldwide developed alternative vaccine platforms. In Poland, the setting of the present study, only two other adenoviral vector vaccines were approved—Vaxzevria (Oxford/AstraZeneca; authorized by the European Medicines Agency [EMA] in January 2021) and Jcovden (Janssen/Johnson & Johnson; EMA authorization granted in March 2021) (7).

Since 2020, studies have shown that Pfizer and Moderna (mRNA-1273) vaccines demonstrate comparable effectiveness against SARS-CoV-2 when administered as a two-dose primary series spaced 3–4 weeks (5). Over time, vaccine efficacy against incident infection waned, driven by viral antigenic evolution and the emergence of variants with escape mutations relative to the ancestral wild-type strain (8).

The effectiveness of vector-type vaccines varies between 92% (for AstraZeneca’s vaccine against the Delta variant when administered in two doses) and 71% (for the single-dose Johnson & Johnson vaccine against the Delta variant when administered in a single dose [as recommended in Poland]). (9)

However, growing public skepticism toward vaccination since the late 1990s has contributed to reduced vaccine acceptance and uptake globally, particularly among children (10, 11). Immunization uptake during pregnancy is even lower (12, 13). Regarding vaccine safety in Poland, the overall vaccine confidence has declined across Europe (especially in the Netherlands, the United Kingdom, the Baltic States, and Eastern Europe). However, there is no comparison between 2020 and 2022 data on the COVID-19 vaccine. New data show that only 68% of Polish respondents find the COVID-19 vaccine safe, compared to 74% of the EU population (9).

### Objective

Our study aimed to assess adherence to compliance with the COVID-19 vaccination recommendations of the Polish Society of Gynecologists and Obstetricians (*Polskie Towarzystwo Ginekologów i Położników-PTGiP*), published in April 2021, among pregnant women hospitalized at the University Clinical Center (UCC) in Gdańsk, Poland (14). Second, age, place of residence, education, and obstetric history were analyzed to identify determinants of higher adherence. Moreover, the study evaluated the prevalence of SARS-CoV-2 infection among women before and during pregnancy. Polish medical reports lack data not only regarding COVID-19 vaccine confidence and adherence among pregnant women, but also COVID-19 morbidity in this population. In the context of declining vaccine confidence, a lack of awareness of the health hazards of COVID-19 infection during pregnancy, and rising anti-vaccine hesitancy, studies such as this one are necessary to address this critical knowledge gap.

## Materials and methods

This cross-sectional study was conducted among postpartum women hospitalized between 18 November 2021 and 28 February 2022, at the Department of Neonatology, University Clinical Center (UCC), Gdańsk, Poland.

Data were collected using a structured questionnaire covering past SARS-CoV-2 infections, COVID-19 vaccination status, demographic characteristics, and obstetric history (subfertility and prior miscarriage). The study included deliveries at both premature and term gestational ages, primiparous and multiparous women, and women with and without a history of pregnancy loss. The inclusion criteria encompassed all hospitalized postpartum women, regardless of age, place of residence, and education level.

The questionnaire was anonymous and voluntary and was administered in a paper-based format; it comprised 12 closed-ended questions (both single- and multiple-choice).

We excluded 14 women due to language barriers with the Polish-language questionnaire, and 2 forms were excluded due to incomplete data.

Ultimately, the study included 675 women hospitalized during the study period who provided informed consent to participate.

Statistical analyses were performed using Statistica version 9.1. Categorical variables were presented as frequencies and percentages, and associations were examined using the chi-square test of independence. A two-sided *p*-value of <0.05 was considered statistically significant. Multivariable logistic regression analysis was performed to evaluate the associations between vaccination status and demographic and clinical variables. The study was conducted in accordance with the Declaration of Helsinki and reported according to the Strengthening the Reporting of Observational Studies in Epidemiology (STROBE) guidelines.

The Ethical Committee at the Medical University of Gdańsk approved the study (NKBBN/367/2021) on 8 April 2021.

## Results

We stratified respondents into subgroups and performed comparative analyses to determine which groups exhibited higher adherence to national vaccination recommendations. Participants’ characteristics are presented in Table 1.

**Table 1:** Patients’ characteristics.

Group characteristic	*n* (%)
Fertility	
Primiparous mothers	344 (51%)
Multiparous mothers	331 (49%)
Domicile	
Large city (>500,000 inhabitants)	358 (53%)
Smaller cities	171 (25.4%)
Countryside	146 (21.6%)
Age	
<19 years	9 (1.3%)
20–25 years	67 (10%)
26–35 years	464 (68.7%)
36–40 years	118 (17.5%)
>40 years	17 (2.5%)
Educational level	
University (>12 years)	468 (69.3%)
Secondary (12 years)	186 (27.6%)
Primary (9 years)	21 (3.1%)
Vaccination against Covid-19 (overall)	
Not vaccinated	287 (42.5%)
Vaccinated before or during pregnancy	388 (57.5%)
Vaccinated against Covid-19 subgroup (*n* = 388)	
Only during pregnancy	238 (61.3%)
Only before pregnancy	57 (14.7%)
Before and during pregnancy	93 (24.0%)

The largest vaccinated group consists of women who were vaccinated only during pregnancy (*n* = 238/675, 35.3%); followed by women vaccinated both before and during pregnancy (*n* = 93/675, 13.8%); only 8.4% (*n* = 57/675) of respondents were vaccinated only before pregnancy, whereas 42.5% (*n* = 287/675) had never received a COVID-19 vaccine.

Among the 150 women vaccinated before pregnancy, 38% did not complete the vaccination course during pregnancy.

Figure 1 illustrates the overall distribution of vaccinated and unvaccinated participants across the groups.

**Figure 1:**
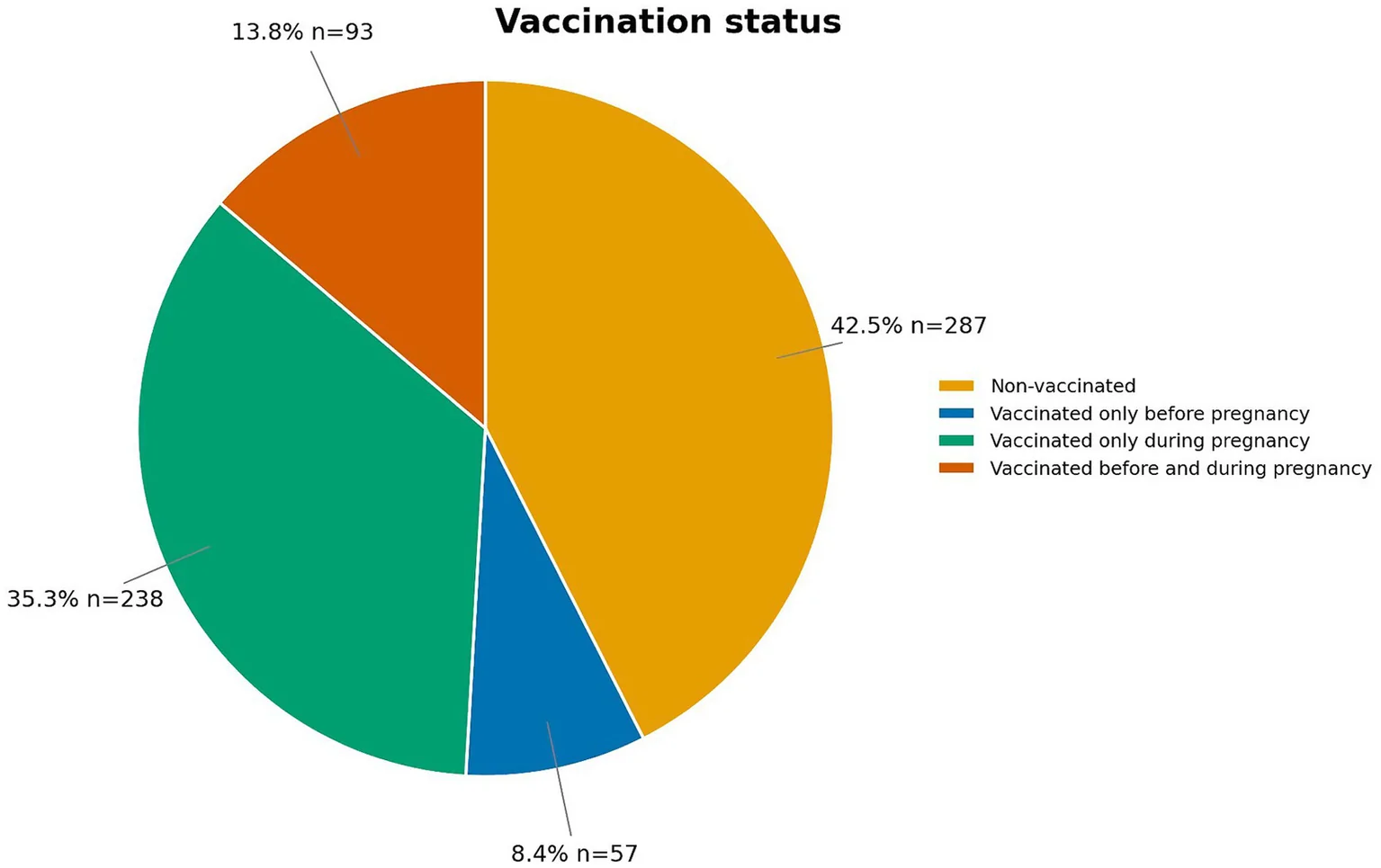
Vaccination status of the respondents.

Among women who received a vaccine dose both before and during pregnancy, the dose administered during pregnancy occurred in the third trimester in 57% of cases (*n* = 53/93), the second trimester in 30.1% (*n* = 28/93), and the first trimester in 19.4% (*n* = 18/93)—the latter contrary to PTGiP recommendations. In six women (6.5%), vaccination during pregnancy was recorded in more than one trimester; the available data do not indicate how many doses this represents. In the group vaccinated only during pregnancy, the majority received vaccination confined to a single trimester: the second trimester in 41.6% of women (*n* = 99/238) and the third trimester in 31.9% (*n* = 76/238). Smaller subgroups had vaccination recorded across two trimesters—second and third in 4.6% (*n* = 11/238), first and second in 6.3% (*n* = 15/238), and first and third in 2.5% (*n* = 6/238)—while 1.7% (*n* = 4/238) had vaccination recorded in all three trimesters. Vaccination confined to the first trimester alone occurred in 11.3% of patients (*n* = 27/238). Overall, 21.8% of women in this group (*n* = 52/238) had at least one vaccination administered during the first trimester, contrary to PTGiP guidelines.

The vast majority of respondents were vaccinated in accordance with the PTGiP recommendations, with the Pfizer vaccine (91.8, 95% CI 88.4–94.3%), while the remaining group received AstraZeneca (4.8, 95% CI 3.0–7.7%), Moderna (2.1, 95% CI 1.0–4.3%), and Johnson & Johnson’s (1.8, 95% CI 0.8–3.9%) vaccines.

Before pregnancy, vaccination distribution differed: 72.7% Pfizer (95% CI 65.0–79.2%); 17.3% AstraZeneca (95% CI 12.1–24.2%); 7.3% Moderna (95% CI 4.1–12.7%); and 3.3% Johnson & Johnson’s (95% CI 1.4–7.6%).

Furthermore, all women vaccinated with Pfizer’s vaccine before pregnancy (*n* = 67) continued with the same vaccine during pregnancy. Among the 93 women vaccinated both before and during pregnancy, 19 (20.4, 95% CI 13.5–29.7%) switched from another vaccine to Pfizer, in accordance with PTGiP recommendations.

During the study, data on COVID-19 infection rates among participants were also collected. Across the entire cohort, 25% (95% CI 21.9–28.4%) of respondents reported being infected before pregnancy, and 12.4% (95% CI 10.2–15.2%) were infected during pregnancy.

The distribution of COVID-19 infections in the “vaccinated during pregnancy” group is slightly different—25.7% (95% CI 21.3–30.7%) were infected before pregnancy, and 8.8% (95% CI 6.2–12.3%) during pregnancy. In the “vaccinated before pregnancy” group, 28.7% (95% CI 22.0–36.4%) were infected before pregnancy, and 14.7% (95% CI 9.9–21.2%) were infected during pregnancy.

We also assessed whether educational attainment influenced willingness to vaccinate. Among respondents vaccinated during pregnancy, 82.5% (95% CI 78.0–86.2%) were university educated, 16.3% (95% CI 12.7–20.7%) had secondary education, and 1.2% (95% CI 0.5–3.1%) had primary education. By contrast, in the unvaccinated group, 56.7% (95% CI 51.4–61.8%) had university education, 38.4% (95% CI 33.4–43.6%) had secondary education, and 4.9% (95% CI 3.1–7.8%) had primary education. These differences were statistically significant (χ^2^(2, *N* = 675) = 53.53, *p* < 0.001, Cramér’s V = 0.28, indicating a small-to-medium effect size).

Furthermore, we found significant differences in pre-pregnancy vaccination status by educational attainment: among women who were not vaccinated before pregnancy, 64.8% (95% CI 60.6–68.7%) had university education, 31.8% (95% CI 28.0–35.9%) had secondary education, and 3.4% (95% CI 2.2–5.4%) had primary education—compared with the vaccinated group (85.3, 95% CI 78.8–90.1% university; 12.7, 95% CI 8.3–18.9% secondary; 2.0, 95% CI 0.7–5.7% primary), χ^2^(2, *N* = 675) = 23.40, *p* < 0.001, Cramér’s V = 0.19 (indicating a small effect size). Among women already vaccinated before pregnancy, we observed no significant differences in educational attainment between those who completed their vaccination course during pregnancy and those who did not (χ^2^(2, *N* = 150) = 0.82, *p* = 0.662, Cramér’s V = 0.07, negligible effect).

We found similar disparities across maternal age categories. Vaccination uptake during pregnancy increased with age: 58.5% (95% CI 50.1–66.5%) for women aged ≥ 36 years, 53.0% (95% CI 46.6–59.4%) for those aged 31–35 years, 44.4% (95% CI 38.2–50.9%) for ages 26–30 years, and 34.2% (95% CI 24.5–45.4%) for women aged <25 years χ^2^(3, *N* = 675) = 14.99, *p* = 0.002, Cramér’s V = 0.15 (small effect size).

Significant differences were also observed when evaluating age in relation to pre-pregnancy vaccination uptake: 34.8% (95% CI 27.3–43.2%) in the ≥36 age group, 20.9% (95% CI 16.2–26.6%) in the 26–30 group, 19.1% (95% CI 14.6–24.7%) in the 31–35 group, and 13.2% (95% CI 7.3–22.6%) in the <25 age group χ^2^(3, *N* = 675) = 17.49, *p* < 0.001, Cramér’s V = 0.16 (small effect size).

To account for the joint effects of place of residence, age, and education on vaccination decisions, we conducted multivariable logistic regression analyses for five definitions of vaccination status.

Forest plots of adjusted odds ratios (aOR) with 95% confidence intervals (95% CI) for sociodemographic predictors of COVID-19 vaccination status in pregnancy, corresponding to the five logistic regression models, are presented in Figure 2.

**Figure 2:**
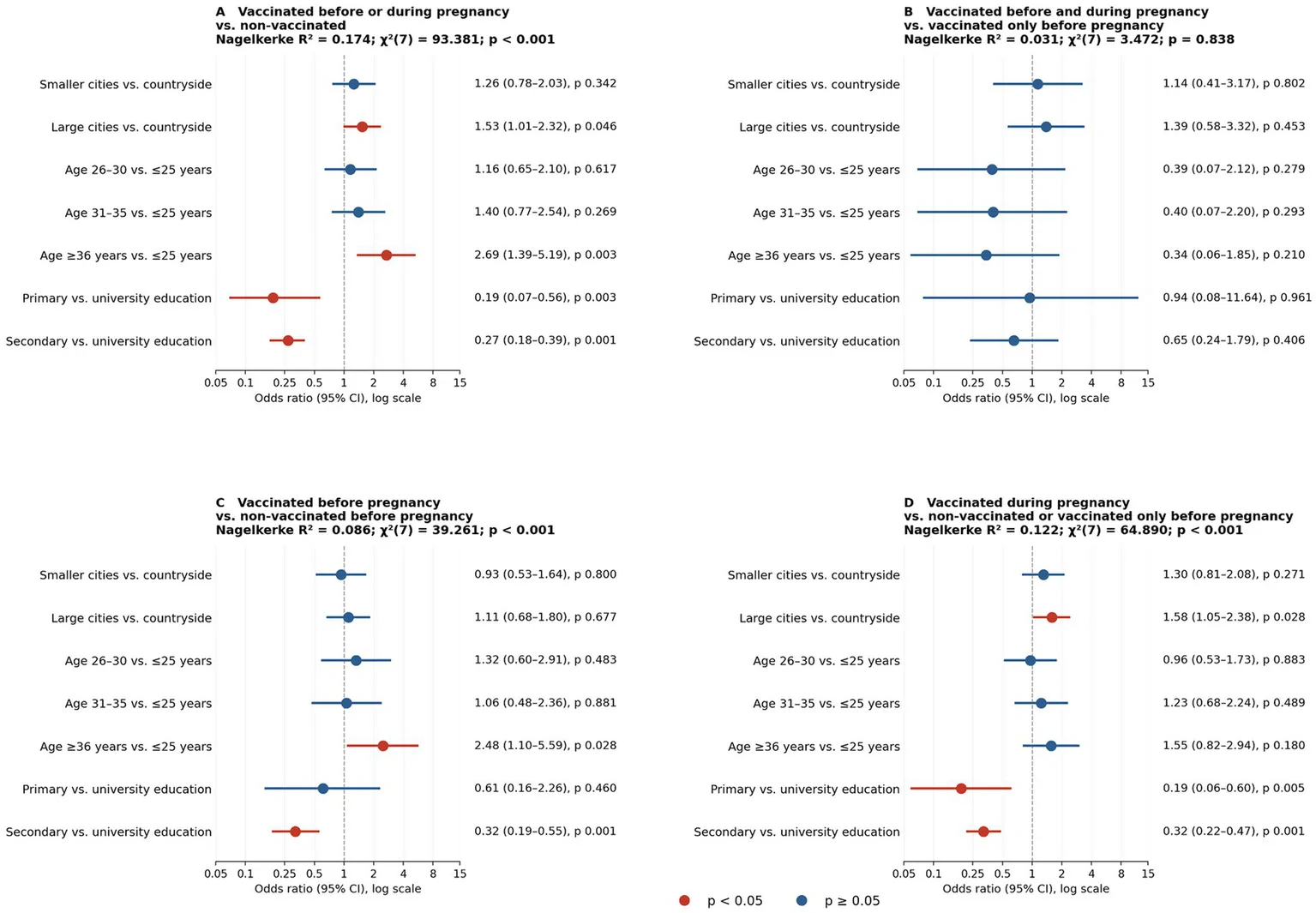
Adjusted odds ratios for the association between socio-demographic characteristics and COVID-19 vaccination status during pregnancy. Forest plots showing adjusted odds ratios (ORs) and 95% confidence intervals (CIs) for the association between socio-demographic characteristics and vaccination status. **(A)** Vaccinated before or during pregnancy versus non-vaccinated. **(B)** Vaccinated before and during pregnancy versus vaccinated only before pregnancy. **(C)** Vaccinated before pregnancy versus non-vaccinated before pregnancy. **(D)** Vaccinated during pregnancy versus non-vaccinated or vaccinated only before pregnancy. Odds ratios are presented on a logarithmic scale. Red markers indicate statistically significant associations (*p* < 0.05), whereas blue markers indicate non-significant associations (*p* < 0.05).

Ever vaccinated before or during pregnancy (*n* = 388) vs. never vaccinated (*n* = 287). This model showed the strongest goodness-of-fit (Nagelkerke *R*^2^ = 0.174, χ^2^(7) = 93.38, *p* < 0.001). Residence in a large city was associated with higher odds of vaccination than in the countryside (OR = 1.529, 95% CI 1.007–2.322, *p* = 0.046). Maternal age ≥36 years was associated with higher odds than <25 years (OR = 2.685, 95% CI 1.388–5.194, *p* = 0.003). Both primary (OR = 0.192, 95% CI 0.066–0.561, *p* = 0.003) and secondary education (OR = 0.266, 95% CI 0.182–0.387, *p* < 0.001) were associated with significantly lower odds than university education.

Vaccinated before and during pregnancy (*n* = 93) vs. vaccinated only before pregnancy (*n* = 57), excluding never-vaccinated women (total *n* = 150). The model was *not* statistically significant overall (Nagelkerke *R*^2^ = 0.031, χ^2^(7) = 3.47, *p* = 0.838), and none of the individual predictors reached statistical significance—i.e., among women vaccinated before pregnancy, place of residence, age, and educational attainment did not distinguish those who continued vaccination during pregnancy from those who did not.

Vaccinated before pregnancy (*n* = 150) vs. not vaccinated before pregnancy (*n* = 525), irrespective of subsequent vaccination during pregnancy. The model was statistically significant (Nagelkerke *R*^2^ = 0.086, χ^2^(7) = 39.26, *p* < 0.001). Place of residence was not statistically significant. Maternal age ≥36 years was associated with higher odds of pre-pregnancy vaccination (OR = 2.480, 95% CI 1.101–5.587, *p* = 0.028). Secondary education was associated with lower odds (OR = 0.322, 95% CI 0.189–0.549, *p* < 0.001), whereas primary education was not a significant predictor (OR = 0.610, *p* = 0.460).

Vaccinated during pregnancy (*n* = 331) vs. not vaccinated during pregnancy (*n* = 344), irrespective of vaccination before pregnancy. The model was statistically significant (Nagelkerke *R*^2^ = 0.122, χ^2^(7) = 64.89, *p* < 0.001). Residence in a large city was associated with higher odds of vaccination during pregnancy (OR = 1.581, 95% CI 1.050–2.378, *p* = 0.028). Maternal age was not statistically significant. Both primary (OR = 0.190, 95% CI 0.060–0.601, *p* = 0.005) and secondary education (OR = 0.322, 95% CI 0.220–0.472, *p* < 0.001) were associated with significantly lower odds of vaccination relative to university education.

Across all four statistically significant models, lower educational attainment was consistently and independently associated with lower odds of vaccination, even after adjusting for place of residence and maternal age—indicating that educational attainment was the strongest and most robust predictor among those tested.

We also evaluated parity (primiparous vs. multiparous) and history of miscarriage; we found no statistically significant differences in vaccination uptake by these obstetric factors.

## Discussion

Historically, the emergence of new pathogens has driven rapid advances in preventive interventions, which have consistently prompted waves of public skepticism and debate regarding vaccine safety and potential adverse effects. This has been accompanied by a rise in public concern and misinformation about vaccine safety and efficacy (15). Insufficient vaccination coverage frequently precipitates localized outbreaks of vaccine-preventable diseases, which could be effectively mitigated through the implementation of mandatory immunization programs (16, 17). Steep declines in vaccine confidence represent a concerning trend, most notable in Eastern and Central Europe, the Baltic states, and the Netherlands (9, 18). Furthermore, the WHO classified Poland as an endemic country for measles due to declining immunization coverage (<95%) (19). Our findings highlight suboptimal COVID-19 vaccination uptake among pregnant women in this cohort, indicating incomplete adherence despite national recommendations. Many women did not follow PTGiP recommendations for COVID-19 vaccination during pregnancy. Most unvaccinated women were younger and had lower educational attainment. In Poland, 22% of women have completed only primary or lower-secondary education, compared with 20% across the broader European Union (20). However, the association between educational attainment and vaccination uptake must be interpreted with caution. Educational attainment is likely closely linked to other determinants of vaccine acceptance, including health literacy, socioeconomic status, access to reliable health information and digital resources, and trust in healthcare professionals and public health institutions. Therefore, the observed association should not be interpreted as causal, but rather as reflecting the complex interplay of multiple sociodemographic and psychosocial factors influencing vaccination decisions.

Our findings suggest that younger pregnant women and those with lower educational attainment represent critical target demographics for tailored vaccine communication during antenatal care. However, educational attainment should not be viewed in isolation as a determinant of vaccine uptake, as it is closely intertwined with health literacy, access to reliable medical information, socioeconomic status, and trust in healthcare providers. Targeted counseling by obstetricians and midwives, together with clear, evidence-based educational initiatives, may help enhance vaccine confidence and uptake in these populations.

More than half of the respondents resided in urban areas. This distribution may reflect higher vaccination uptake among women in urban settings, potentially driven by better access to healthcare services, specialized antenatal care, and reliable health information. However, this observation should be interpreted cautiously, as the study was not specifically powered or designed to investigate the underlying structural drivers of urban–rural disparities (21–23).

This study was conducted among women hospitalized at a single university tertiary center in a large Polish city, which may limit the generalizability of the findings to the broader population of pregnant women in Poland. In addition, only 20% of respondents reported living in rural areas, suggesting that the study sample may underrepresent rural populations and not fully capture disparities in their willingness to receive vaccination.

Mathieu et al.’s study (24) reported that, as of 8 February 2026, Poland documented 3,152 cumulative confirmed COVID-19 deaths per million people. This rate exceeded mortality figures observed across Europe (2,823 per million) and worldwide (893 per million). Furthermore, as of 12 August 2024, an average of 171,88 vaccine doses had been administered per 100 people globally. Worldwide, more than 70% of the population had received at least one dose, whereas in Poland, as of December 2023, 60% had received at least one dose.

In our study, approximately 50% of pregnant women received a COVID-19 vaccine, with the majority (91.8%) receiving the Pfizer vaccine, in line with PTGiP recommendations. Notably, vaccine uptake was higher among older women, with the highest rates in the “≥ 36 years” group. The “State of Vaccine Confidence in the EU (2022)” report also found that younger respondents (aged 18–34) were less likely than those in the older age group (over 65 years) to agree that vaccines are important, safe, and effective. The “vaccine confidence” gap between younger and older respondents is widening, particularly in Belgium, Bulgaria, Croatia, France, Hungary, Latvia, Poland, and the Netherlands (9). Overall, between 2018 and 2022, vaccine confidence in Europe rose more in the ≥65 age group than in the 18–34 age group. These data are highly concerning, as vaccine confidence in the younger group is directly related to the successful delivery of routine childhood immunization programs worldwide.

### Limitations

This study has several limitations to consider when interpreting its findings. First, the study was conducted among women hospitalized at a single university tertiary center in a large Polish city, which may limit the generalizability of the results to the broader population of pregnant women in Poland. Furthermore, only 20% of participants reported living in rural areas, suggesting that the sample may not adequately represent the geographical distribution of pregnant women in Poland or potential disparities in attitudes toward vaccination across urban and rural settings.

Another limitation concerns the observed association between educational attainment and vaccination uptake. Although women with higher educational attainment were more likely to be vaccinated, this association should not be interpreted as a direct causal effect. Educational attainment may reflect a broader set of sociodemographic and contextual characteristics, including socioeconomic status, access to healthcare services, health literacy (the capacity to obtain and evaluate health-related information), and trust in medical professionals. Furthermore, the exclusion of women unable to complete the Polish-language questionnaire may have introduced selection bias, limiting the generalizability of the findings to non-Polish-speaking populations. Because these potential confounding factors were not comprehensively assessed in the present study, residual confounding cannot be ruled out. Future studies incorporating a wider range of social and behavioral variables are warranted to clarify the mechanisms underlying the relationship between education and vaccination decisions.

## Conclusion

Since 2025, the Polish Chief Sanitary Inspectorate has expanded vaccination recommendations to include routine COVID-19 vaccination for all pregnant women. The recommended vaccinations currently include influenza, respiratory syncytial virus (RSV), diphtheria–pertussis–tetanus (Tdap), and COVID-19 (25). Consequently, perinatal and neonatal infection rates are anticipated to decline over the coming years. Our findings indicate that lower educational attainment was the most consistent sociodemographic factor associated with lower COVID-19 vaccination uptake among pregnant women in this cohort. Older maternal age and residence in a large city were also associated with higher odds of vaccination in selected multivariable analyses. These findings support the need for tailored, evidence-based vaccine communication during antenatal care, particularly for women with lower educational attainment. Further multicenter studies incorporating socioeconomic factors, health literacy, access to healthcare, and attitudes toward vaccination are warranted to better understand the determinants of maternal vaccine uptake. Recent studies demonstrate that higher educational attainment promotes greater trust in medical expertise, leading to improved adherence to medical recommendations and higher vaccination uptake (26, 27).

## Data Availability

The raw data supporting the conclusions of this article will be made available by the authors, without undue reservation.
